# Infrared broadband metasurface absorber for reducing the thermal mass of a microbolometer

**DOI:** 10.1038/s41598-017-00586-x

**Published:** 2017-03-27

**Authors:** Joo-Yun Jung, Kyungjun Song, Jun-Hyuk Choi, Jihye Lee, Dae-Geun Choi, Jun-Ho Jeong, Dean P. Neikirk

**Affiliations:** 1 Nano-convergence Mechanical Systems Research Division, Korea Institute of Machinery and Materials, 156 Gajeongbuk-Ro, Daejeon, 305-343 Republic of Korea; 2 Microelectronics Research Center, Department of Electrical and Computer Engineering, The University of Texas at Austin, Austin, TX 78712-1024 USA

## Abstract

We demonstrate an infrared broadband metasurface absorber that is suitable for increasing the response speed of a microbolometer by reducing its thermal mass. A large fraction of holes are made in a periodic pattern on a thin lossy metal layer characterised with a non-dispersive effective surface impedance. This can be used as a non-resonant metasurface that can be integrated with a Salisbury screen absorber to construct an absorbing membrane for a microbolometer that can significantly reduce the thermal mass while maintaining high infrared broadband absorption in the long wavelength infrared (LWIR) band. The non-dispersive effective surface impedance can be matched to the free space by optimising the surface resistance of the thin lossy metal layer depending on the size of the patterned holes by using a dc approximation method. In experiments a high broadband absorption was maintained even when the fill factor of the absorbing area was reduced to 28% (hole area: 72%), and it was theoretically maintained even when the fill factor of the absorbing area was reduced to 19% (hole area: 81%). Therefore, a metasurface with a non-dispersive effective surface impedance is a promising solution for reducing the thermal mass of infrared microbolometer pixels.

## Introduction

There are two major classes of infrared detection: photon detection and thermal detection. Photon detectors generally provide a faster response time than thermal detectors^1^ but usually require a cryogenic cooling system to prevent the excessive thermal generation of charge carriers^2^. One of the main obstacles to the widespread use of infrared systems based on semiconductor photon detectors are the cooling requirements. In contrast to photon detection, uncooled bolometric thermal detection relies on the increase in temperature of the thermally isolated bolometric material caused by the absorption of incident infrared radiation, which can then be detected by the temperature-induced change in the electrical resistance. Historically, the Salisbury screen absorber, which consists of a resistive absorber layer with a non-dispersive surface impedance (~377 Ω) placed a quarter-wavelength in front of a metal mirror layer, has been used for broadband microbolometers in the long wavelength infrared (LWIR) band. However, the response speed of an infrared microbolometer may be slow because of the very high thermal resistance required for sensitivity. In other words, the absorbing membrane of microbolometer pixels, which is composed of a resistive absorber layer, bolometric layer, and mechanical support layer, is suspended over the metal mirror layer using high thermal resistance arms to provide thermal isolation. The typical values of the thermal time constant, which determines the response speed of microbolometers, for commercial microbolometers are 15 and 7 ms corresponding to the vanadium oxide microbolometer and the amorphous silicon microbolometer, respectively^2–4^. The thermal time constant is proportional to the thermal mass of the absorbing membrane of microbolometer pixel^2, 3^. Therefore, the only way to increase the response speed is to reduce the thermal mass, which is proportional to the mass of the absorbing membrane. One way to reduce the thermal mass is by using a thinner absorbing membrane. For mechanical support, however, microbolometers require a finite thickness support layer; this limits how much the thermal mass of the microbolometer can be reduced. Another way to reduce the thermal mass is by making patterned holes in the absorbing membrane, but this reduces the fill factor of the absorbing layer.

Recently, patterning periodic subwavelength-sized holes in a metal layer or metal elements on a substrate have been shown to produce unique spectral responses through the abrupt changes in the phase and amplitude of the incident light at the surface of the metal layer for various electromagnetic wave bands from microwaves to the optical wavelengths^5–13^. These two-dimensional structures are known as metasurfaces. They can be treated as thin layers with an equivalent effective surface impedance and have been used as part of narrowband^14^ or tunable absorbers^15–20^. These metasurface absorbers have near-unity absorption, even though the fill factor of the absorbing area of metasurface is small. However, the spectral responses of these metasurface absorbers show wavelength-selective narrowband absorption because the effective surface impedance of metasurfaces having the nature of a resonator that is extremely dispersive around the resonance wavelength. In addition, there are some approaches to develop wavelength-selective microbolometers using the resonant property of metasurface^14^ and metamaterial absorber^21–23^.

In this study, we demonstrated that a large fraction of periodic patterned holes in a thin lossy metal layer with optimised surface resistance can produce an absorbing membrane for a microbolometer that significantly reduces the thermal mass while maintaining high infrared absorption over a wide range of wavelengths. This can be accomplished by incorporating a non-resonant metasurface characterised with a non-dispersive effective surface impedance into a classical Salisbury screen absorber to form an asymmetric Fabry–Perot cavity. We experimentally determined that, even with a fill factor for the absorbing area reduced to 28% (hole area: 72%), the metasurface absorber can absorb as much infrared energy as the Salisbury screen absorber in the LWIR band. Our proposed metasurface absorber can provide an efficient solution for reducing the thermal mass of the absorbing membrane of a microbolometer.

## Results and Discussions

### Design of the non-resonant metasurface

**Figure 1:**
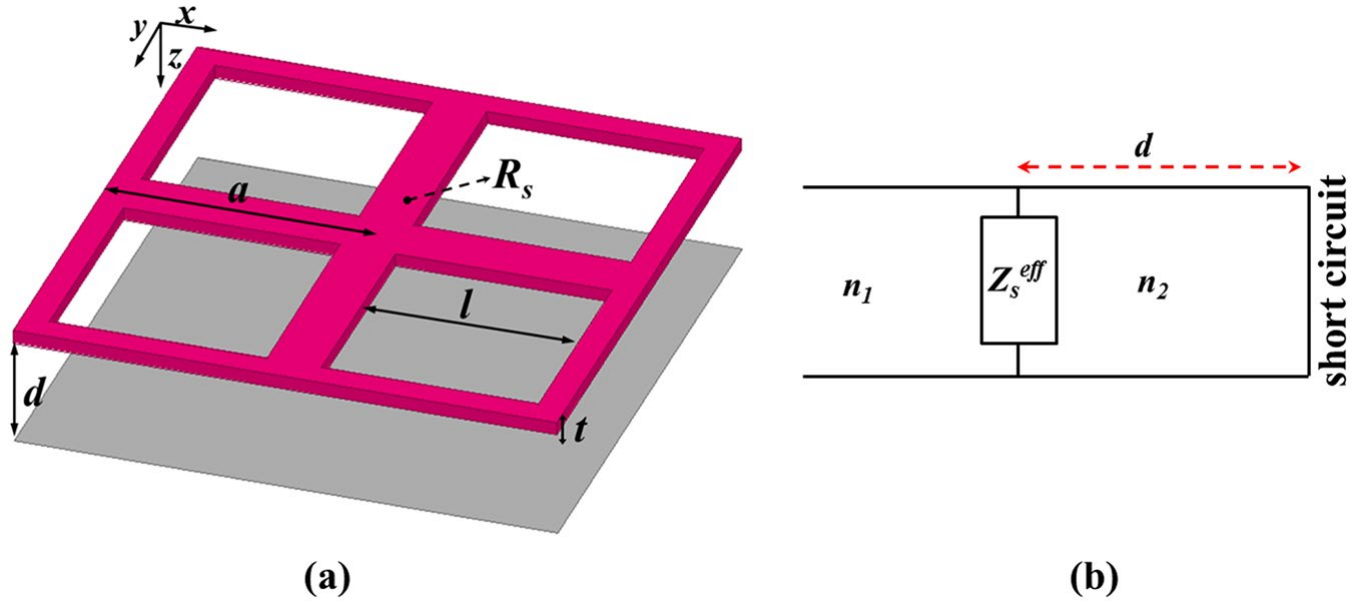
(**a**) Schematic of a metasurface absorber layer. (**b**) Equivalent transmission line.

Figure 1 shows a schematic and the transmission line model of the proposed metasurface absorber. The overall metasurface absorber has four basic design parameters: the array period *a*, vacuum gap distance *d* to the metal mirror, side length *l* of a square, and thickness *t* of the metasurface. At some wavelength, an electric resonance induced by the structure dominates the spectral response of the metasurface and determines the dispersive effective surface impedance. Therefore, one important strategy for obtaining a non-dispersive effective surface impedance is to use the non-resonant metasurface at a wavelength range far away from the resonance wavelength. We assumed that the thickness of the metasurface is less than the skin depth *δ* and free space operating wavelength *λ* (i.e. *t* < *δ* ≪ *λ*). Therefore, the metasurface can be treated as an absorbing resistive surface that is characterised by the effective surface impedance. A lossy metal with frequency-independent (dc) resistivity (*ρ*) in the LWIR band would be an ideal choice for an absorbing resistive surface with a non-dispersive effective surface impedance. The effective surface impedance for a metasurface to achieve broadband absorption can be engineered according to the side length *l* of a square and surface resistance (*R*_*s*_ = *ρ*/*t*) of the lossy metal metasurface. Finding the optimal surface resistance for a certain hole size is critical to obtaining the non-dispersive effective surface impedance that produces broadband absorption in the LWIR band. Because the size of the patterned holes and the array period *a* are much smaller than that of the operating wavelength, the electromagnetic behaviour of the structure can be described by a quasi-static approximation (dc approximation), and the surface current density distribution can be determined by the design parameters of the metasurface.

**Figure 2:**
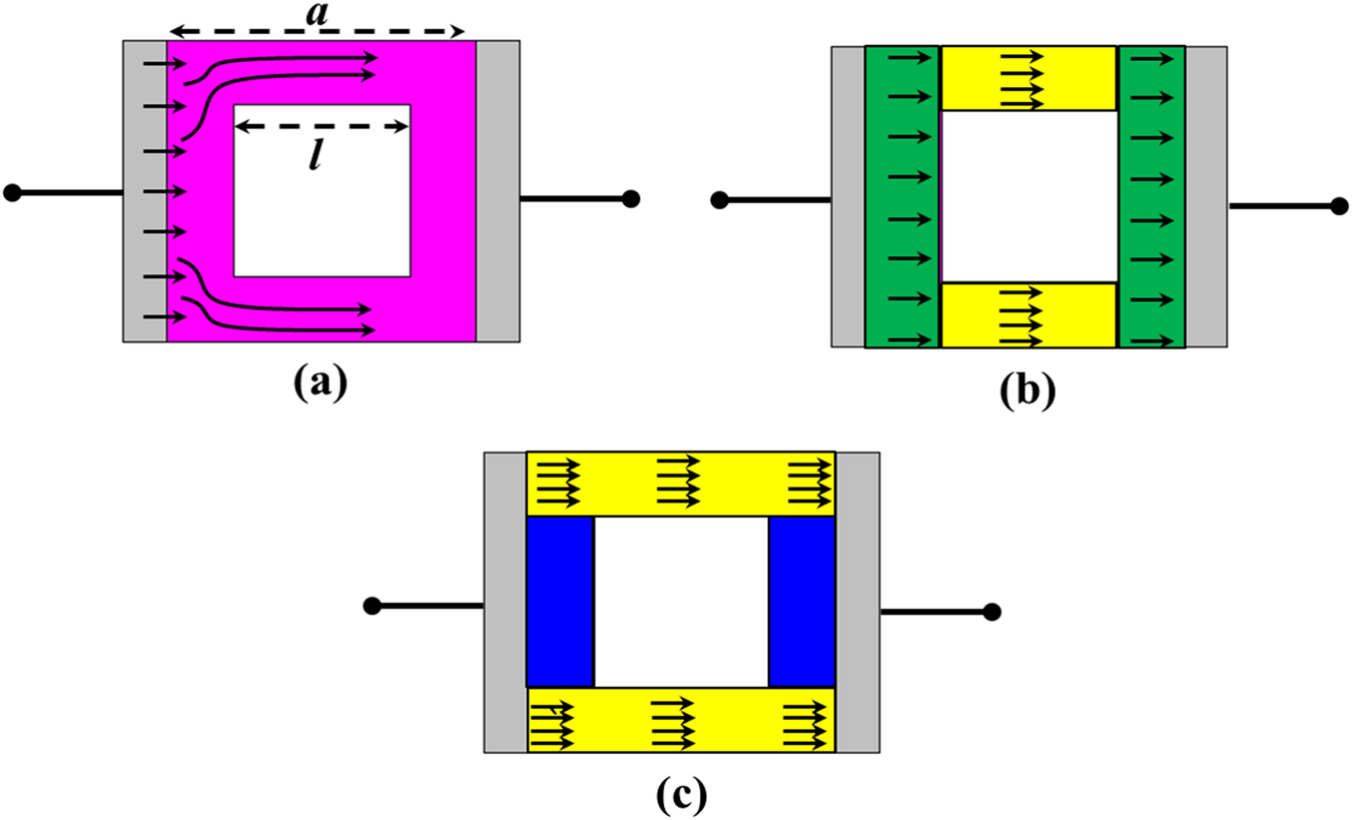
(**a**) Illustration of the current redistribution around the corners of the surface. (**b**) Lower bound case: the surface resistance of both ends (green areas) is *R*_s_(*a* − *l*)/*a*, and the surface resistance of the two side sheets (yellow areas) is *R*_*s*_ *l*/(*a* − *l*). (c) Upper bound case: the surface resistance of the two long sheets (yellow area) is *R*_s_ *a*/(*a* − *l)* with no surface current in the blue area.

Figure 2 shows the surface resistance dc approximation for a single unit cell of a square hole patterned metasurface for use when the array period *a* is much smaller than the wavelength. A metallic surface with holes causes surface current crowding on the surface, as illustrated in Fig. 2(a). There are two simple estimates to account for surface current crowding on such a surface: setting a lower bound, as shown in Fig. 2(b), and setting an upper bound, as shown in Fig. 2(c). In the case of the lower bound, the surface current is assumed to flow on both ends (indicated by the green shading in Fig. 2(b)) and two sides of the sheet (indicated by the yellow shading in Fig. 2(b)). The analytical formula of the surface resistance for such a current distribution is

1 $${R}_{lower}={R}_{s}(\frac{a-l}{a}+\frac{l}{a-l})\,$$

In the case of the upper bound, the surface current is assumed to flow on just two long sheets (indicated by the yellow shading in Fig. 2(c)). The analytical formula of the surface resistance for such a current distribution is

2 $${R}_{upper}={R}_{s}(\frac{a}{a-l})$$

For example, if we set the array period *a* = 1 μm, which is smaller than the wavelength for the LWIR band (roughly 8–14 μm), and the side length of the square hole *l* = 0.7 μm, the fill factor of the surface is reduced by 51% (hole area: 49%). For a normal Salisbury screen, the surface impedance of the resistive absorber layer is purely resistive (*R*_*s*_ = 377 Ω) to obtain perfect absorption. Setting *R*_*lower*_ and *R*_*upper*_ = 377 Ω allows the necessary surface resistance *R*_*s*_ for the square hole patterned metasurface to be estimated. For these example dimensions, the surface resistance from the lower bound is 143 Ω, while the surface resistance from the upper bound is 113 Ω.

### Effective surface impedance of the metasurface

To verify this dc approximation, numerical methods and formula to retrieve the effective surface conductivity (*σ*_*s*_^*eff*^ = 1/*Z*_*s*_^*eff*^) of the thin metal and graphene metasurface were used to calculate the effective surface impedance of the metasurface^14–20^. The effective surface conductivity of a metasurface can be expressed as^19^

3 $${\sigma }_{s}^{eff}{Z}_{0}=\frac{2}{{S}_{21}}\sqrt{{n}_{1}{n}_{2}}-({n}_{1}+{n}_{2})$$

This proposed formula for the retrieved effective surface conductivity is representative for asymmetric dielectric layers. In this work, we only consider the normal incidence of electromagnetic waves on the metasurface in a symmetric environment (i.e. the metasurface in a free space) since the absorption at normal incidence is highly dominant due to the relatively low NA lens used in microbolometer imaging systems. Therefore, *n*_1_ and *n*_2_ are the refractive indices of the free space. *Z*_0_ is the characteristic impedances of the free space. The *S*_21_ parameter is related to the transmission coefficient for a wave coming from the *n*_1_ side of the free space.

**Figure 3:**
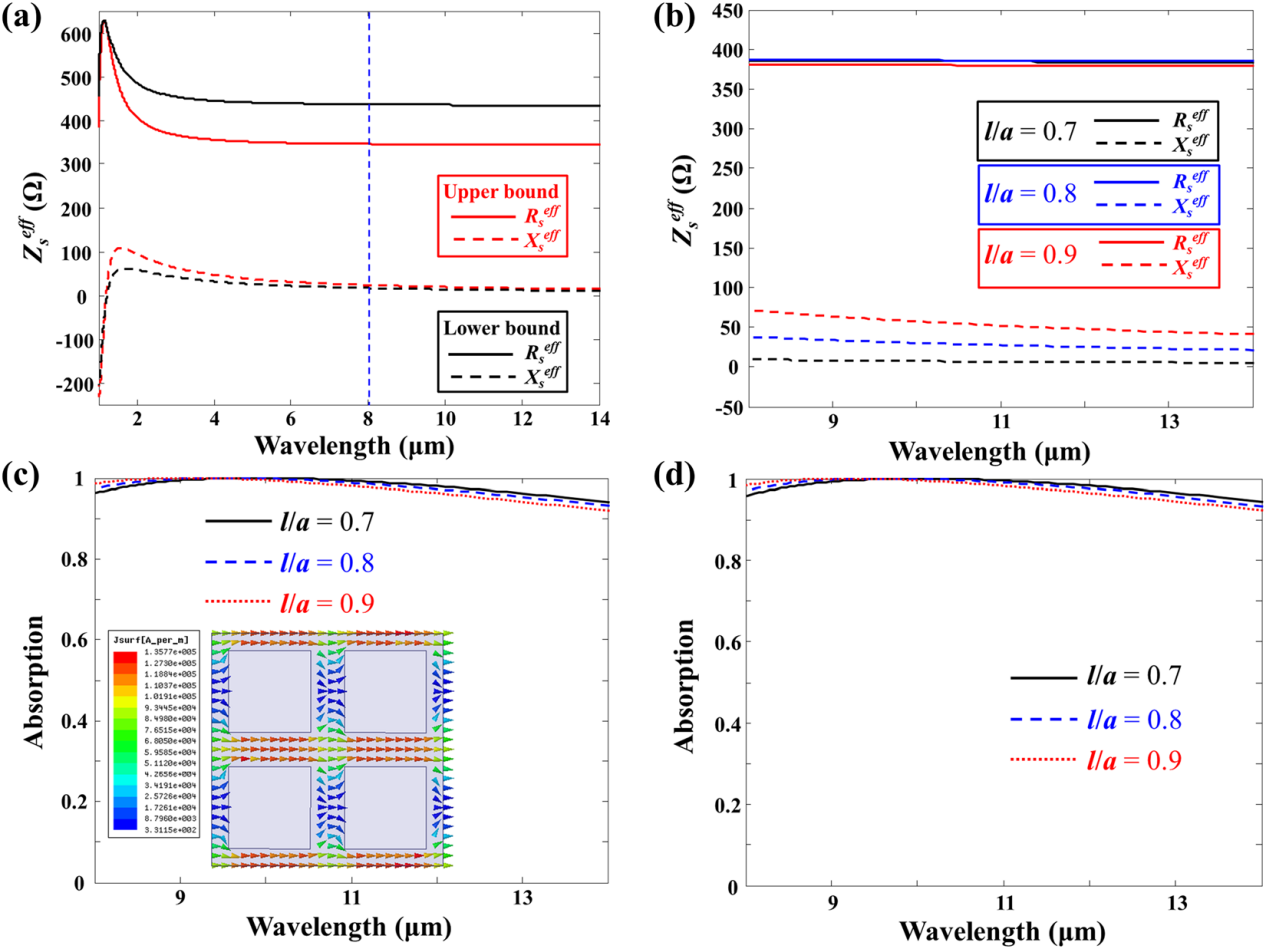
(**a**) Retrieved effective surface impedances of the metasurfaces for both the upper bound (red curves) and lower bound (black curves) cases. (**b**) Retrieved effective surface impedances of the metasurfaces for *l* = 0.7 μm with *R*_*s*_ = 128 Ω (black curves), *l* = 0.8 μm with *R*_*s*_ = 83 Ω (blue curves), and 0.9 μm with *R*_*s*_ = 40 Ω (red curves). (**c**) Calculated absorption spectral responses of the metasurface absorbers for *l* = 0.7 μm with *R*_*s*_ = 128 Ω (black curves), *l* = 0.8 μm with *R*_*s*_ = 83 Ω (blue curves), and 0.9 μm with *R*_*s*_ = 40 Ω (red curves) with identical vacuum gap distances of *d* = 2.5 μm. The inset shows the simulated surface current density distribution on the metasurface with *l* = 0.7 μm at the resonance wavelength. (**d**) Simulated absorption spectral responses of the metasurface absorbers for *l* = 0.7 μm with *R*_*s*_ = 128 Ω (black curves), *l* = 0.8 μm with *R*_*s*_ = 83 Ω (blue curves), and 0.9 μm with *R*_*s*_ = 40 Ω (red curves) with identical vacuum gap distances of *d* = 2.5 μm.

Figure 3(a) shows the retrieved effective surface impedance of the metasurface in both the upper bound case (*R*_*s*_ = 113 Ω) and lower bound case (*R*_*s*_ = 143 Ω) for the surface resistance with the side length of the square hole *l* = 0.7 μm. As expected, both the real and imaginary parts of the retrieved effective impedance in both cases were nearly constant over the LWIR band far away from the resonance wavelength induced by the patterned square hole array. The effective surface impedances of the metasurface for the upper and lower bound cases were about 340 + 10*j*  Ω and 429 + 2.5 *j* Ω, respectively. To achieve better absorption (i.e. find an effective surface impedance similar to *Z*_*s*_^*eff*^ = 377 Ω), the surface resistance still needed to be optimised between the lower and upper bounds. The average surface resistance of the lower and upper bounds was determined to be the optimised surface resistance. As shown in Fig. 3(b), the retrieved surface impedance (*Z*_*s*_^*eff*^ = 384.6 + 7.3 *j* Ω) of the metasurface with the optimised surface resistance *R*_*s*_ = 128 Ω approached the ideal surface impedance (*Z*_*s*_^*eff*^ = 377 Ω) and was constant over the LWIR band. In addition, the retrieved effective surface impedances of the metasurfaces for bigger patterned hole cases (*l* = 0.8 and 0.9 μm) with different optimised surface resistances are presented in Fig. 3(b). The effective surface impedances (*Z*_*s*_^*eff*^ = 386 + 31.78 *j* Ω for *l* = 0.8 μm and *Z*_*s*_^*eff*^ = 380.3 + 63 *j* Ω for *l* = 0.9 μm) of metasurfaces for both *l* = 0.8 μm with *R*_*s*_ = 83 Ω and 0.9 μm with *R*_*s*_ = 40 Ω were non-dispersive in the LWIR band, and the real part of the effective surface impedances for both cases approached the ideal surface impedance. The imaginary part of the effective surface impedances was observed to become larger as the dimensions of the square patterned holes were increased. Figure 3(c) and (d) show the calculated absorption spectral responses using transmission line theory with the retrieved effective surface impedances and simulated absorption spectral responses using the numerical simulation HFSS for three different patterned hole sizes of metasurface absorbers with the identical vacuum gap distance *d* = 2.5 μm, which was a quarter of the wavelength of 10 μm. The calculated results were in excellent agreement with the simulated results and showed perfect broadband absorptions (99.99% at a wavelength of 9.87 μm, 99.89% at a wavelength of 9.49 μm, and 99.97% at a wavelength of 9.09 μm) in the LWIR band despite the fill factor of the absorbing area being reduced to 49%, 36%, and 19% corresponding to *l* = 0.7 μm, *l* = 0.8 μm, and *l* = 0.9 μm, respectively.

In our previous work^14^, the following analytical solution was presented for the required ideal surface impedance of a metasurface with perfect absorption as a function of the effective thickness of the spacer layer (*dn*_2_/*λ*) between the metasurface and bottom metal mirror layer:

4 $${Z}_{s}^{eff}=\frac{j{Z}_{0}{Z}_{d}\,\tan ({\beta }_{d}d)}{j{Z}_{d}\,\tan ({\beta }_{d}d)-{Z}_{0}}$$

where *Z*_*d*_ *=* *Z*_0_ */n*_2_ is the characteristic impedance of the spacer layer and *β*_*d*_ = (2*πn*_2_)/*λ* is the propagation constant of the spacer layer. The analytical solution offers that the ideal effective surface impedances of each metasurface should be 376.7 + 7.7 *j* Ω at a wavelength of 9.87 μm, 374 + 31.6 *j* Ω at a wavelength of 9.49 μm, and 367.6 + 58.3 *j* Ω at a wavelength of 9.09 μm when the thickness of the spacer layer is 2.5 μm. The retrieved effective surface impedances of each metasurface shown in Fig. 3(b) are almost identical to the ideal effective surface impedances for perfect absorption. Therefore, these metasurface absorbers achieved near-unity absorption at the resonance wavelength, as shown in Fig. 3(c). Also, the larger imaginary part of the effective surface impedance as the dimensions of the square patterned holes of the metasurface increased caused the absorption peak to shift to a shorter wavelength, as shown in Fig. 3(c) and (d). To verify the surface current density distribution on the metasurface in our two assumed simple cases, the surface current density distribution on the metasurface with *l* = 0.7 μm at the resonance wavelength was determined, as shown in the inset of Fig. 3(c). The surface current distribution for the entire square patterned hole surface was similar to the lower bound case, as shown in Fig. 2(b). However, the amount of surface current on both ends was much smaller than the surface current flowing on the two long sides of the surface in the upper bound case, as shown in Fig. 2(c). Even when the fill factor was reduced to 19% (hole area: 81%), the metasurface absorber absorbed nearly as much as the Salisbury screen absorber with *R*_*s*_ = 377 Ω.

### Experimental results and discussion

**Figure 4:**
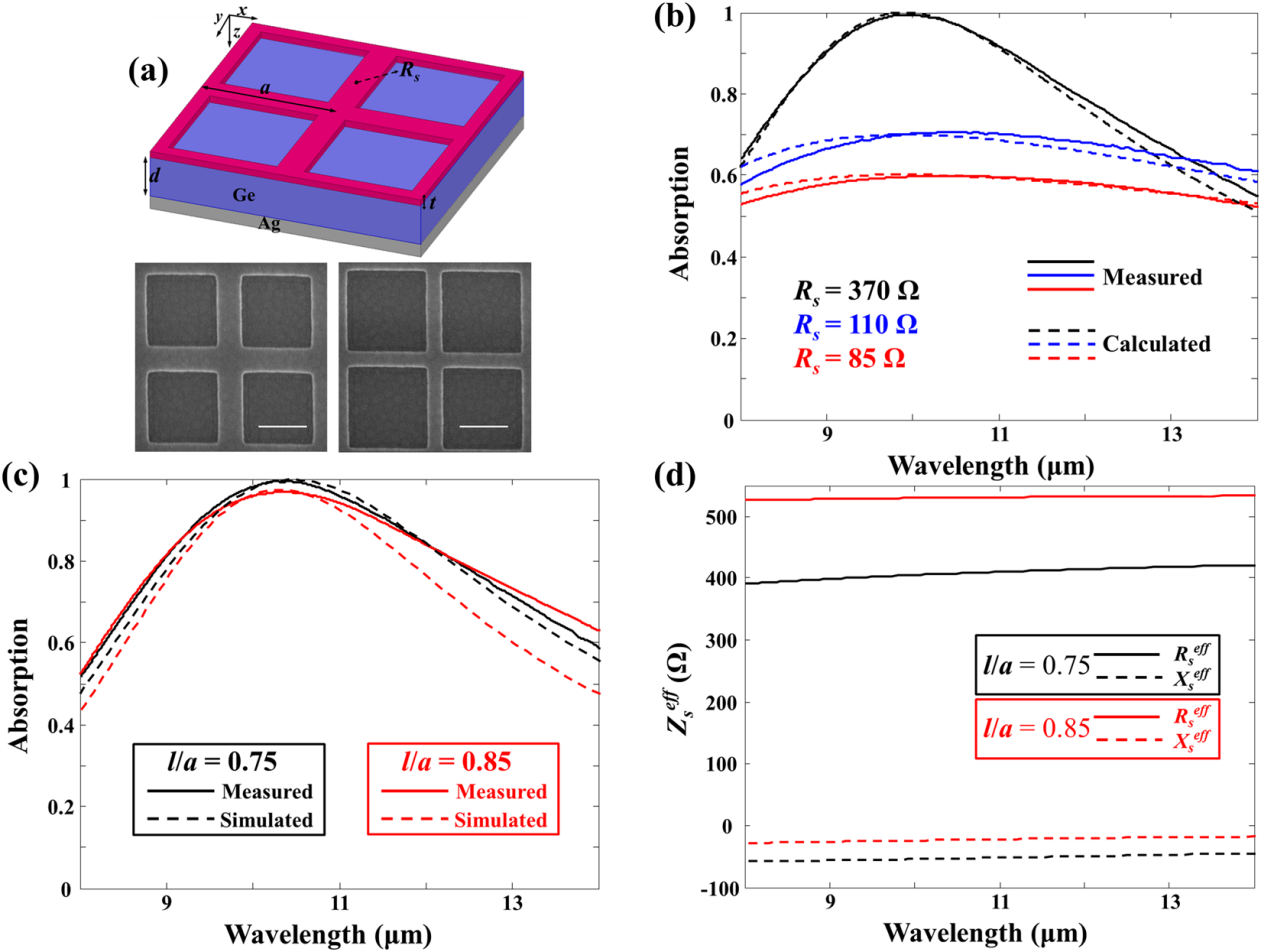
(**a**) Schematic and SEM images of the fabricated metasurface absorbers. Scale bar is 500 nm. (**b**) Measured (solid curves) and calculated (dashed curves) absorption spectral responses of Salisbury screen absorbers with three different surface resistances: *R*_*s*_ = 85 (red curves), 110 (blue curves), and 370 Ω (black curves). (**c**) Measured (solid curves) and simulated (dashed curves) absorption spectral responses of the metasurface absorbers for *l* = 0.75 μm with *R*_*s*_ = 110 Ω (black curves) and *l* = 0.85 μm with *R*_*s*_ = 85 Ω (red curves). (**d**) Retrieved effective surface impedances of the metasurfaces for *l* = 0.75 μm with *R*_*s*_ = 110 Ω (black curves) and *l* = 0.85 μm with *R*_*s*_ = 85 Ω (red curves).

Fabricating a free-standing thin metal layer with a periodic hole array is not easy. To overcome this difficult fabrication issue and demonstrate the proposed idea, we chose germanium (Ge), which is almost lossless and has a nearly constant refractive index of *n* = 4 in the LWIR band^24^, and a spacer layer instead of a vacuum gap between the metasurface and bottom metal mirror layer. For the parallel type a quarter-wavelength resonator such as Salisbury scree absorber, the working bandwidth (*BW*~1/*ωRC*) is inversely proportional to the capacitance increased due to the high refractive index of Ge spacer layer^25, 26^. Therefore, a high refractive index of Ge spacer layer causes the working bandwidth of the metasurface absorber to decrease. The metasurface absorbers with different patterned hole sizes (*l* = 0.75 and 0.85 μm) in Fig. 4(a) were fabricated by using e-beam lithography and e-beam evaporator deposition processes.

To obtain the designed surface resistance of the absorbing resistive layer, determining a proper lossy metal with dc resistivity in LWIR band was critical. However, most of the metals in the LWIR band, which is a high frequency region (*ωτ* > 1), show frequency-dependent optical properties, which can be fit using Drude model with the plasma frequency *ω*_*p*_ and the damping constant *γ* = 1/*τ* ^27^. The damping constant of the thin metal layer is higher than that of the bulk metal due to the surface scattering and the grain boundary effect in the thin metal layer^28, 29^. Therefore, the increased damping constant of a high-resistivity thin metal layer causes the LWIR band to extend to a low frequency region (*ωτ* < 1). Nichrome (NiCr) is a high-resistivity metal and was used as absorbing resistive layer in the LWIR band^30–32^. The optical constants of NiCr as thin absorbing layer, which satisfy the condition of a low frequency region (*ωτ* ≪ 1), followed the Hagen-Rubens relation with *n* = *k* = 3.61 $\sqrt{\lambda \,}$ ^30^. In addition, the optical constants of thin NiCr film in the LWIR band showed the difference dependent on the thickness and the detail of fabrication^31, 32^. As a result, using a high-resistivity NiCr as the absorbing resistive layer is not critical in the LWIR band. NiCr for the fabricated metasurface absorbers was assumed to have dc resistivity in the LWIR band. This was confirmed by using calibration structures, which were simple unpatterned Salisbury screen absorbers with three different surface resistances of *R*_*s*_ = 85, 110, and 370 Ω corresponding to absorbing resistive layers of *t* = 35, 25, and 12 nm, respectively. The dc surface resistance of the NiCr absorbing resistive layer was measured by using a four-point probe. Figure 4(b) compares the absorption spectral responses measured with FTIR and calculated with the transmission line theory. The measured results with three different surface resistances agreed well with the calculated results, which confirmed that the surface resistances of the NiCr absorbing resistive layer in the LWIR band behaved as the dc surface resistances. Figure 4(c) shows the measured (solid curves) and simulated (dotted curves) absorption spectral responses of two different fabricated absorbers with *l* = 0.75 μm and *R*_*s*_ = 110 Ω (black curves) and with *l* = 0.85 μm and *R*_*s*_ = 85 Ω (red curves), which agreed reasonably well with each other.

As shown in Fig. 4(d), the retrieved effective surface impedances of both metasurfaces with *l* = 0.75 μm and *R*_*s*_ = 110 Ω (black curves) and with *l* = 0.85 μm and *R*_*s*_ = 85 Ω (red curves) were non-dispersive in the LWIR band as expected. The optimised surface resistances for the metasurfaces with *l* = 0.75 μm and 0.85 μm according to our proposed dc approximation method were around *R*_*s*_ = 105 and 60 Ω, respectively. Therefore, even when the fill factor was reduced to 44% (hole area: 56%), a fabricated metasurface absorber with *l* = 0.75 μm and *R*_*s*_ = 110 Ω, which was similar to the optimised surface resistance *R*_*s*_ = 105 Ω, absorbed (99.5% absorption with the working bandwidth over 90% absorption of 2.18 μm) as much as the Salisbury screen absorber with *R*_*s*_ = 370 Ω (99.4% absorption with the working bandwidth, over 90% absorption of 2.16 μm), as shown in Fig. 4(b). Also, a fabricated absorber with *l* = 0.85 μm and *R*_*s*_ = 85 Ω produced fairly broad absorption (96.9% absorption with the working bandwidth, over 90% absorption of 2.02 μm) in the LWIR band even when the fill factor was reduced to 28% (hole area: 72%). The optimised surface resistance of the metasurfaces with *l* = 0.85 μm should be around *R*_*s*_ = 60 Ω corresponding to NiCr absorbing resistive layer of *t* = 65 nm. The increased thickness of the absorbing resistive layer would increase the thermal mass of the absorbing membrane.

**Figure 5:**
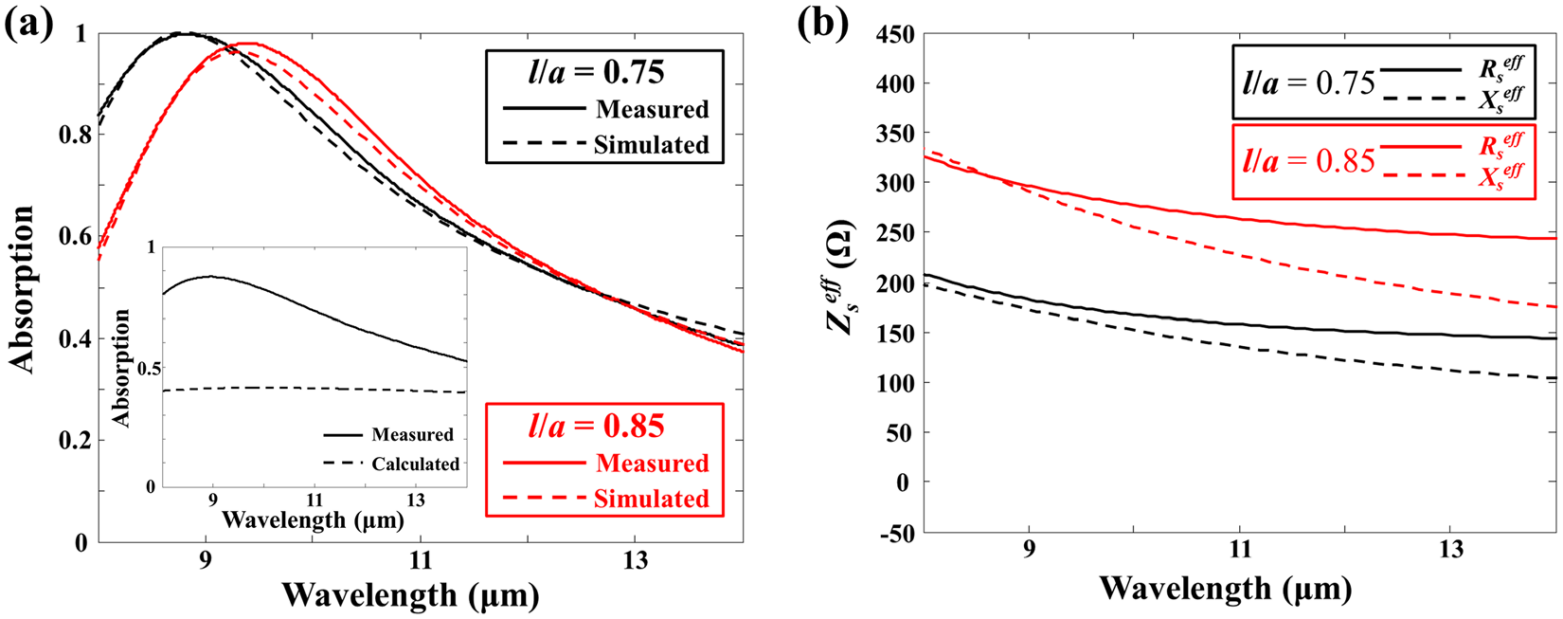
(**a**) Measured (solid curves) and simulated (dashed curves) absorption spectral responses of the metasurface absorbers for *l* = 0.75 μm (black curves) and *l* = 0.85 μm (red curves) with identical Ni thicknesses of *t* = 12 nm. The inset shows the measured (black solid) absorption spectral response of Salisbury screen absorbers with a Ni thickness of *t* = 12 nm and calculated (black dashed) absorption spectral responses of Salisbury screen absorbers with *R*_*s*_ = 50 Ω. (**b**) Retrieved effective surface impedances of the metasurfaces for *l* = 0.75 μm (black curves) and *l* = 0.85 μm (red curves) with identical Ni thicknesses of *t* = 12 nm.

Thus, in order to obtain a low surface resistance such as *R*_*s*_ = 60 Ω, nickel (Ni) was used and assumed to have dc resistivity in LWIR band. Again, for calibration, a simple unpatterned Salisbury screen absorber with the surface resistance *R*_*s*_ = 50 Ω (*t* = 12 nm), which was measured by using a four-point probe, was characterised with FTIR, as shown in the inset of Fig. 5(a). The comparison between the measured and calculated absorption spectral responses confirmed that the surface resistances of the Ni absorbing resistive layer in LWIR band did not behave as the dc surface resistances. The permittivity of the bulk Ni in the LWIR band can be described by the Drude model with the plasma frequency *ω*_*p*_ = 7.41 × 10^15^ rad/s and the damping constant *γ* = 6.66 × 10^13^ rad/s^27^. Because of the effect of the grain boundary and the surface scattering in the thin metal layer, the damping constant of the thin Ni layer was higher than that of the bulk Ni^28, 29^. Figure 5(a) compares the measured absorption spectral responses (solid curves) and simulated absorption spectral responses (dotted curves) with three times the damping constants of Ni for metasurface absorbers with both *l* = 0.75 and 0.85 μm and identical Ni thicknesses of *t* = 12 nm. The measured results were in excellent agreement with the simulated results. Experimentally, we achieved an absorption of 99.65% with the working bandwidth and over 90% absorption of 1.51 μm for the metasurface absorber with *l* = 0.75 μm. We also achieved an absorption of 97.7% with the working bandwidth and over 90% absorption of 1.29 μm for the metasurface absorber with *l* = 0.85 μm. Both of the metasurface absorbers produced reasonably good broadband absorption in the LWIR band.

As shown in Fig. 5(c), the retrieved effective surface impedances of the metasurfaces with the Ni absorbing resistive layer were not as constant as those of the metasurfaces with the NiCr absorbing resistive layer because the permittivity of Ni in the LWIR band is dispersive and described by the Drude model. Therefore, the absorption spectral responses of the metasurface absorbers with the Ni absorbing resistive layer exhibited less broadband absorption than those of the metasurface absorbers with the NiCr absorbing resistive layer. In addition, the absorption peaks of both metasurface absorbers shifted the short wavelength. The retrieved effective surface impedances of the metasurfaces with *l* = 0.75 μm and 0.85 μm were 187.2 + 178 *j* Ω at a wavelength of 8.77 μm and 288.9 + 278.7 *j* Ω at a wavelength of 9.32 μm. From the analytical solution, the ideal effective surface impedances of each metasurface for the perfect absorption should be 221.8 + 185.4 *j* Ω at a wavelength of 8.77 μm and 323.5 + 131.3 *j* Ω at a wavelength of 9.32 μm. Because the retrieved effective surface impedance of the metasurface with *l* = 0.75 μm was similar to the ideal effective surface impedance of the metasurface for the perfect absorption at a wavelength of 8.77 μm, the metasurface absorber achieved near-unity absorption at the wavelength of 8.77 μm, as shown in Fig. 5(b). However, the difference between the ideal and retrieved effective surface impedances of the metasurface with *l* = 0.85 μm indicates that the metasurface absorber only produced 96% absorption at the wavelength of 9.32 μm.

From the experimental and simulated data, the metasurface absorbers with the NiCr absorbing resistive layer produce more broadband absorption than the metasurface absorbers with the Ni absorbing resistive layer. However, from the perspective of thermal mass reduction, the metasurface absorbers with the Ni absorbing resistive layer are much attractive. The thermal mass of metal absorbing resistive layer is the proportional to the mass and the heat capacity of a specific metal, and the density and the heat capacity of NiCr and Ni are almost identical^33^. Therefore, the thermal mass reduction in absorbing resistive layer is determined by the volume reduction in absorbing resistive layer. The metasurface absorber with the Ni absorbing resistive layer and *l* = 0.85 μm has three times less thermal mass than the metasurface absorber with the NiCr absorbing resistive layer and *l* = 0.75 μm even with the working bandwidth is reduced to almost 60%. This concept of the metasurface absorber is applicable to microbolometer pixels. A large fraction of periodic patterned holes in the thermally isolated absorbing membrane of microbolometer pixels can reduce the thermal mass without decreasing the IR absorption. In addition, real microbolometer pixels would contain a finite array of unit cells, in contrast to the assumption of an infinite array of unit cells in the numerical simulation. Decreasing the array size of the metasurface to within the size of device operating wavelength would induce the resonance wavelength to shift to a shorter wavelength and the amplitude of the absorption to decrease^34^. When the array size of the metamaterial absorber is larger than the device operating wavelength, the metamaterial absorber still exhibits excellent absorption^21^. Therefore, the size of microbolometer pixels containing a finite array of unit cells for our proposed metasurface should be larger than the device operating wavelength (14 μm), which is comparable with the pixel size (<20 μm) of current microbolometers.

## Conclusion

We demonstrated that infrared broadband metasurface absorbers can reduce the thermal mass of microbolometer pixels by incorporating a metasurface with a non-dispersive effective surface impedance into a Salisbury screen absorber. The retrieved non-dispersive effective surface impedance matched to the free space can be achieved by finding the optimised dc surface resistance of a thin lossy metal for a certain patterned hole size by using the proposed dc approximation method. Experiments with the fabricated metasurface absorbers showed outstanding broadband absorption in the LWIR band, even when the fill factor of the absorbing area was reduced to 27.75%. Therefore, this concept for the metasurface absorber can be used in current state-of-the-art uncooled infrared thermal detectors.

## Method

### Sample fabrication and characterization

The fabrication of the metasurface absorbers started with depositing 10/150 nm chromium (Cr)/silver (Ag) as the bottom metal mirror layer on a silicon substrate. This was followed by the deposition of a 620 nm Ge spacer layer on the Ag mirror layer. After the e-beam lithography process, a proper lossy metal with a proper thickness was deposited and lifted off. The fabricated metasurface absorbers were measured by using a Fourier transform infrared spectroscopy (FTIR) microscope (Bruker Hyperion 3000) in reflectance mode at a resolution of 4 cm^−1^.

### Numerical simulations

The *S*_21_ parameter was simulated by using the commercial numerical software HFSS. The normal incidence of LWIR radiation polarised parallel to the *x*-direction was used as the excitation source from the top of a single unit of the metasurface with a periodic boundary condition in the lateral direction. Because the thickness of the metasurface was much smaller than the operating wavelength, we used a patterned infinitely thin layer with a proper surface resistance as the metasurface in the simulation.
